# The immunosuppression pathway of tumor‐associated macrophages is controlled by heme oxygenase‐1 in glioblastoma patients

**DOI:** 10.1002/ijc.34270

**Published:** 2022-09-15

**Authors:** Sara Magri, Beatrice Musca, Laura Pinton, Elena Orecchini, Maria Laura Belladonna, Ciriana Orabona, Camilla Bonaudo, Francesco Volpin, Pietro Ciccarino, Valentina Baro, Alessandro Della Puppa, Susanna Mandruzzato

**Affiliations:** ^1^ Department of Surgery, Oncology and Gastroenterology University of Padova Padova Italy; ^2^ Immunology and Molecular Oncology Veneto Institute of Oncology IOV—IRCCS Padova Italy; ^3^ Department of Medicine and Surgery University of Perugia Perugia Italy; ^4^ Neurosurgery, Department of NEUROFARBA University of Florence, University Hospital of Careggi Florence Italy; ^5^ Neurosurgery Department University Hospital of Padova Padova Italy; ^6^ Academic Neurosurgery, Department of Neurosciences University of Padova Padova Italy

**Keywords:** glioblastoma, heme oxygenase‐1, iron metabolism, macrophages, tumor microenvironment

## Abstract

The immunosuppressive tumor microenvironment (TME) in glioblastoma (GBM) is mainly driven by tumor‐associated macrophages (TAMs). We explored whether their sustained iron metabolism and immunosuppressive activity were correlated, and whether blocking the central enzyme of the heme catabolism pathway, heme oxygenase‐1 (HO‐1), could reverse their tolerogenic activity. To this end, we investigated iron metabolism in bone marrow‐derived macrophages (BMDMs) isolated from GBM specimens and in in vitro‐derived macrophages (Mφ) from healthy donor (HD) blood monocytes. We found that HO‐1 inhibition abrogated the immunosuppressive activity of both BMDMs and Mφ, and that immunosuppression requires both cell‐to‐cell contact and soluble factors, as HO‐1 inhibition abolished IL‐10 release, and significantly reduced STAT3 activation as well as PD‐L1 expression. Interestingly, not only did HO‐1 inhibition downregulate *IDO1* and *ARG‐2* gene expression, but also reduced IDO1 enzymatic activity. Moreover, T cell activation status affected PD‐L1 expression and IDO1 activity, which were upregulated in the presence of activated, but not resting, T cells. Our results highlight the crucial role of HO‐1 in the immunosuppressive activity of macrophages in the GBM TME and demonstrate the feasibility of reprogramming them as an alternative therapeutic strategy for restoring immune surveillance.

## Graphical abstract

What's new?

Macrophages are vital for normal iron homeostasis and contribute to dysregulated iron metabolism in the tumor microenvironment. In glioblastoma, macrophages also play a role in immunosuppression. This study shows that the immunosuppressive activity of bone marrow‐derived macrophages (BMDMs) in glioblastoma is linked to iron metabolism. Inhibition of heme oxygenase‐1 (HO‐1), the central enzyme of the heme catabolism pathway, abolished the immunosuppressive activity of BMDMs. This effect simultaneously modulated other downstream inhibitory mechanisms, including IL‐10 and IDO1 and ARG2, which are involved in l‐arginine and l‐tryptophan catabolism. Targeting HO‐1 is a promising strategy for restoring immune surveillance in the tumor microenvironment.

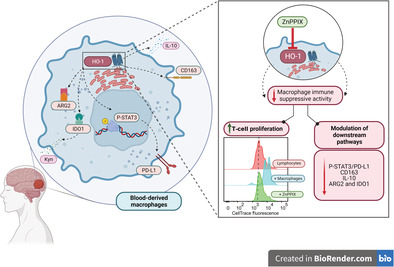

**Abbreviations**
1‐MT: 1‐methyltryptophan
5‐ALA: 5‐aminolevulinic acid
ARG‐1: arginase 1
BMDM: bone marrow‐derived macrophage
CoPPIX: cobalt protoporphyrin IX
FC: fold change
FFPE: formalin‐fixed paraffin‐embedded
GBM: glioblastoma
GFAP: glial fibrillary acidic protein
HD: healthy donor
HO‐1: heme oxygenase‐1
IDO1: indoleamine 2,3‐dioxygenase 1
Kyn: l‐kynurenine
LNC: lipid nanocapsule
M‐CSF: macrophage colony‐stimulating factor
MG: microglia
mIHC: multiplexed immunohistochemistry
Mφ: in vitro‐derived macrophages
Nor‐NOHA: Nω‐hydroxy‐nor‐l‐arginine
PBMC: peripheral blood mononuclear cell
PD‐L1: programmed death ligand 1
PPIX: protoporphyrin IX
SnPPIX: Tin protoporphyrin IX
TAM: tumor‐associated macrophage
TME: tumor microenvironment
ZnPPIX: zinc protoporphyrin IX

## 1 INTRODUCTION

Targeting the tumor microenvironment (TME) is currently considered to be a potential alternative for subverting tumor progression and inducing effective antitumor immunity.^1^ For example, TME signals generally skew macrophages toward a tumor‐promoting phenotype that fosters different aspects of tumor progression, from cell proliferation and genetic instability to angiogenesis and metastasis.^2^, ^3^, ^4^, ^5^ For this reason, high macrophage infiltration has been associated with a poor patient prognosis in different solid tumors^6^ and targeting these cells appears to be a likely strategy to block tumor advance.

In the TME, the establishment of a highly immunosuppressive microenvironment by tumor‐associated macrophages (TAMs) is pivotal for tumor progression, and is achieved through the release of mediators with immunosuppressive or antiinflammatory activity (such as IL‐10 or TGF‐β), the expression of immune checkpoints (such as programmed death ligand 1, PD‐L1) or the recruitment of other immunosuppressive cells (including T regulatory cells, Tregs),^3^ thus providing a protective niche to support tumor growth. Changes in the metabolic pathways of TAMs also contribute to the development of the immunosuppressive microenvironment: modifications in amino acid metabolism are, for example, one of the strategies used by macrophages to starve T cells and to create an unfavorable setting for antitumor responses to succeed. In this respect, the roles of arginase‐1 (ARG‐1) and indoleamine 2,3‐dioxygenase 1 (IDO1), involved in the catabolism of l‐arginine and l‐tryptophan, respectively, have been clearly established in the immunosuppressive network in cancer.^7^

Given macrophages' critical function in iron homeostasis, it is not surprising that such cells play a role in the context of TME, where neoplastic cells have a high demand for iron to support their proliferation, and they tend to adopt an iron‐releasing phenotype.^8^, ^9^ As a result, iron metabolism appears to be dysregulated in several cancer settings.^10^

In line with these considerations, researches conducted in our laboratory in the context of glioblastoma (GBM) revealed that the major immune population infiltrating this tumor is composed of bone marrow‐derived macrophages (BMDMs). In addition, 5‐aminolevulinic acid (5‐ALA), the prodrug administered to patients to guide tumor resection, is mainly metabolized by BMDMs.^11^ Since its metabolic product protoporphyrin IX (PPIX) is a heme precursor, we hypothesized that these cells maintain a sustained iron‐recycling metabolism, which was confirmed by single‐cell RNA sequencing studies on BMDMs, microglia (MG) and tumor cells from GBM samples.^11^

In light of BMDMs' strong immunosuppressive activity in GBM and their iron‐recycling phenotype, we investigated the correlation between these two pathways. The aim of our study was to explore the downstream effects of heme oxygenase‐1 (HO‐1), the rate‐limiting enzyme in the heme catabolism pathway, on the immune suppressive functions of human tumor‐associated BMDMs, found upregulated in our previous study.^11^ Because of its potent antioxidant and antiinflammatory properties,^12^ HO‐1 inhibition was already exploited as a strategy to reduce tumor growth and metastases formation, as well as an emerging immunoregulatory target.^13^, ^14^, ^15^, ^16^, ^17^

Our results establish the central role of HO‐1 in macrophage‐mediated immune suppression present in the glioblastoma microenvironment, and suggest the possibility of reprogramming these cells in the TME by exploiting the iron metabolic pathway in order to restore antitumor cytotoxic immunity and, eventually, boost immunotherapy.

## 2 MATERIALS AND METHODS

### 2.1 Patient characteristics and samples

Patients were enrolled at the Department of Neurosurgery of the Padova and Florence University Hospitals. Freshly resected tumor specimens were included in our study with a confirmed diagnosis of grade IV GBM (as defined by the 2016 WHO classification) by standard histopathological and molecular analyses. When 5‐ALA‐assisted surgery was employed, tissue specimens were derived from the PPIX bright fluorescent area, corresponding to the central non‐necrotic area.^11^ In all other cases, resected specimens originated from the central non‐necrotic area of GBM.

### 2.2 Isolation of BMDMs and MG from GBM tissues

Tumor samples were either processed on the same day of resection or kept at 4°C in MACS Tissue Storage Solution (Miltenyi Biotec, Bergisch Gladbach, Germany) and processed the following day. GBM specimens were washed with 0.9% sodium chloride to remove peripheral blood traces and then digested mechanically and enzymatically by using Tumor Dissociation Kit (Miltenyi Biotec) and gentleMACS Octo Dissociator (Miltenyi Biotec) according to the manufacturer's instructions for soft tumors. Single‐cell suspensions were counted and stained with LIVE/DEAD Fixable Aqua (Life Technologies, Thermo Fisher Scientific, Waltham, Massachusetts), anti‐CD45 BV421 (BD Biosciences, Becton Dickinson, Franklin Lakes, New Jersey), anti‐CD33 PE‐Cy7 (eBioscience, Thermo Fisher Scientific, Waltham, Massachusetts), anti‐HLA‐DR APC (BD Biosciences) and anti‐CD49d PE (BioLegend, San Diego, California). In the case of cell sorting, the single‐cell suspension was subjected to either immunomagnetic bead‐based separation or fluorescence‐activated cell sorting (FACS) to isolate BMDMs and MG, as previously described.^11^

### 2.3 In vitro differentiation of macrophages

Peripheral blood mononuclear cells (PBMCs) were isolated from buffy coats of healthy donors (HDs) using a density gradient centrifugation on Ficoll‐Paque PLUS (GE Healthcare‐Amersham, Buckinghamshire, UK). Monocytes were separated from lymphocytes by adhesion and cultured in 24‐well plates for 7 days with 100 ng/mL M‐CSF (Miltenyi Biotec) (see Supplementary Methods in Appendix S1).

### 2.4 Multiplexed immunohistochemistry analysis

A multiplexed immunohistochemistry (mIHC) analysis was performed on formalin‐fixed paraffin‐embedded (FFPE) tissue slides using the Mantra multispectral imaging platform (Akoya Biosciences, Marlborough, Massachusetts). FFPE slides were stained with anti‐CD8 (clone C8/144B, Thermo Fisher Scientific), anti‐CD68 (clone PG‐M1, Dako Agilent, Santa Clara, California), anti‐HO‐1 (clone HO‐1‐1, Thermo Fisher Scientific) and anti‐glial fibrillary acidic protein (GFAP, clone EP672Y, Abcam, Cambridge, UK) antibodies. DAPI (Akoya Biosciences) was used as a nuclear counterstain. The autofluorescence background signal was subtracted by using an unstained control slide processed in parallel. The Mantra multispectral imaging platform (Akoya Biosciences) was employed for image acquisition at ×20 magnification and data analysis was carried out with InForm 2.4.1 software (Akoya Biosciences).

### 2.5 Cell treatments

BMDMs and Mφ were treated with 10 μM OB 24 hydrochloride (Tocris Bioscience, Bio‐Techne SRL, Minneapolis, Minnesota), 10 μM SnPPIX (Tin Protoporphyrin IX, Tocris Bioscience) and 5 μM ZnPPIX (Zinc Protoporphyrin IX, Tocris Bioscience) at 37°C, 5% CO_2_ (30 minutes for BMDMs and 1 hour for Mφ). Mφ were also pretreated with 5 μM CoPPIX (Cobalt Protoporphyrin IX, Tocris Bioscience) for 1 hour at 37°C, 5% CO_2_ and with 10 μM Stattic (Merck KGaA, Darmstadt, Germany) for 30 minutes at room temperature. The proper drug concentrations were established by analyzing cell viability after staining with LIVE/DEAD Fixable Aqua (Life Technologies, Thermo Fisher Scientific) (data not shown). Treatments were performed in RPMI medium supplemented with 10% fetal bovine serum (FBS, Gibco, Thermo Fisher Scientific), 10 mM Hepes Buffer, 100 U/mL penicillin/streptomycin, 0.28 mM asparagine, 1.5 mM glutamine, 0.67 mM arginine and with the selected drugs, and completely removed before subsequent evaluations. Conversely, the treatment with 1 mM 1‐methyltryptophan (1‐MT, Sigma‐Aldrich) or 0.5 mM Nω‐Hydroxy‐nor‐l‐arginine (Nor‐NOHA, Merck KGaA) was added in co‐cultures.

### 2.6 PD‐L1, CD163 and HO‐1 flow cytometry evaluation

Mφ were stained with anti‐CD163 PerCP‐Cy5.5 (clone GHI/61, BD Biosciences) and anti‐PD‐L1 PE (clone MIH1, eBioscience, Thermo Fisher Scientific) antibodies 24 hours after treatment with the different HO‐1 inhibitors/inducer to analyze the expression of these surface markers by flow cytometry. Fc Receptor (FcR) Blocking Reagent (Miltenyi Biotec) diluted 1:25 with PBS 1% FBS was used to block nonspecific binding sites.

For HO‐1 intracellular staining, Mφ were detached with cold PBS and incubated with BD Cytofix/Cytoperm Fixation and Permeabilization Solution (BD Biosciences) for 20 minutes at 4°C. After washing with PBS enriched with 0.5% bovine serum albumin (BSA, Sigma‐Aldrich) and 0.1% saponin (Sigma‐Aldrich), Mφ were stained with anti‐HO‐1 FITC mAb (clone HO‐1‐2, Abcam) in PBS supplemented with 4% FBS for 30 minutes at 4°C.

### 2.7 Immunosuppressive activity assay

The immunosuppressive activity of BMDMs and in vitro‐derived Mφ, untreated or pretreated with the different HO‐1 inhibitors or treated in co‐culture with ARG‐1 and IDO1 inhibitors, was determined by assessing the proliferation of allogeneic CellTrace‐labeled PBMCs as detailed in the Supplementary Methods in Appendix S1 and as previously described.^11^ Suppression was calculated by analyzing the absolute number of proliferating cells using TruCount tubes (BD Biosciences) that permit the quantitative measurement of cells. Data were normalized by assuming the proliferation of T cells cultured alone as 100%.

### 2.8 Real‐time qPCR

Total cell RNA was isolated from cell pellets before and after treatments. The spectrophotometer NanoDrop (Implen GmbH, Munich, Germany) was used to assess RNA quantity and purity, and a reverse transcription reaction was performed with the Veriti Thermal Cycler (Applied Biosystems, Thermo Fisher Scientific) with SuperScript II Reverse Transcriptase (Invitrogen, Thermo Fisher Scientific). Real‐time polymerase chain reaction (real‐time PCR) was performed using LightCycler 480 Probes Master kit (Hoffmann‐La Roche, Basel, Switzerland) and specific probes (TaqMan Gene Expression Assays, Applied Biosystem) for the genes of interest, with the LightCycler 480 II instrument (Hoffmann‐La Roche).

### 2.9 Western blot analysis

Mφ were lysed for 40 minutes at 4°C in cold RIPA buffer containing 150 mM NaCl (Sigma‐Aldrich), 1% NP‐40 (Sigma‐Aldrich), 0.5% sodium deoxycholate (Sigma‐Aldrich), 0.1% sodium dodecyl sulfate (SDS, Invitrogen) and 50 mM Tris (Sigma‐Aldrich), pH 8.0, with 1x protease inhibitor cocktail (set III, Calbiochem, Sigma‐Aldrich) and 1x phosphatase inhibitor cocktail (P5726, Sigma‐Aldrich). The lysates were centrifuged at 15 000*g* at 4°C for 15 minutes and the supernatants were run on 10% gradient Bis‐Tris SDS‐PAGE Gels (Thermo Fisher Scientific) (40 μg of proteins per lane). Separated proteins were transferred to a nitrocellulose membrane and immunoblotted for specific antibodies according to the manufacturer's instructions. The following primary antibodies were used: mouse anti‐HO‐1 mAb (HO‐1‐1, Abcam); rabbit anti‐STAT3 and anti‐p‐STAT3 (Tyr705) mAbs (Cell Signaling Technology, Danvers, Massachusetts); mouse anti‐α‐tubulin mAb (Santa Cruz Biotechnology); rabbit anti‐β‐actin mAb (Sigma‐Aldrich). The following secondary antibodies were used: goat anti‐mouse IgG horseradish peroxidase‐ (HRP‐) labeled mAb (Perkin Elmer); goat anti‐rabbit IgG HRP‐labeled mAb (Perkin Elmer). Chemiluminescence was developed with SuperSignal West Pico Chemiluminescent Substrate (Thermo Fisher Scientific). The signal was acquired on a ChemiDoc XRS System (Bio‐Rad, Hercules, California) and analyzed with QuantityOne 1D analysis software (Bio‐Rad) and ImageJ software (National Institutes of Health, Bethesda, Maryland). Each protein band was quantified and then normalized after subtracting the background to its loading control.

### 2.10 IL‐10 secretion assay

Mφ, untreated or treated with 5 μM ZnPPIX, were added to FBS‐coated polypropylene tubes for 14 hours at 37°C, 5% CO_2_, with T cells in the presence of BD GolgiPlug Protein Transport Inhibitor (BD Biosciences) containing Brefeldin A, according to the manufacturer's instructions. Treatment with 1 mM 1‐MT was directly added in co‐culture for the entire period of incubation with T cells. Autologous T cells were thawed and activated with 1 μg/mL anti‐CD3 and 5 μg/mL anti‐CD28 mAbs for 24 hours at 37°C, 5% CO_2_. After 14 hours of contact, cell cultures were fixed and permeabilized by using PerFix‐nc Kit (Beckman Coulter, California) according to the manufacturer's instructions and stained for 1 hour with anti‐CD3 PE‐Cy7 (Beckman Coulter) and anti‐IL‐10 BB700 (BD Biosciences) mAbs.

### 2.11 Flow cytometry analysis

Flow cytometry data acquisition was carried out with a BD LSRII flow cytometer (BD Biosciences). For data analysis, FlowJo software (BD Biosciences) was used.

### 2.12 Kynurenine determination

The enzymatic activity of IDO1 was measured in vitro in terms of cell ability to metabolize tryptophan to l‐kynurenine (Kyn), whose concentration was measured in culture supernatants using HPLC as described in.^18^ Briefly, a sample volume of 300 μL was eluted by a mobile phase containing 10 mM NaH_2_PO_4_ pH 3.0 (99%) and methanol (1%) (Sigma‐Aldrich, Missouri), at a flow rate of 1 mL/min, on a Kinetex C18 column (250 × 4.6 mm, 5 μm, 100 Å; Phenomenex). Kyn was detected at 360 nm using a Perkin Elmer Series 200 HPLC instrument (Massachusetts). The detection limit of the assay was 0.05 μM.

### 2.13 Statistics

Mann‐Whitney *U*‐test or *t*‐test was used to evaluate statistically significant variations between groups of samples. Pearson correlation was carried out to test the association between parameters. Differences were considered statistically significant with *P* ≤ .05. All statistical analyses were performed with the SigmaPlot software (Systat Software Inc., San Jose, California).

## 3 RESULTS

### 3.1 HO‐1 expression and localization in macrophage cell subsets present in GBM TME

**FIGURE 1:**
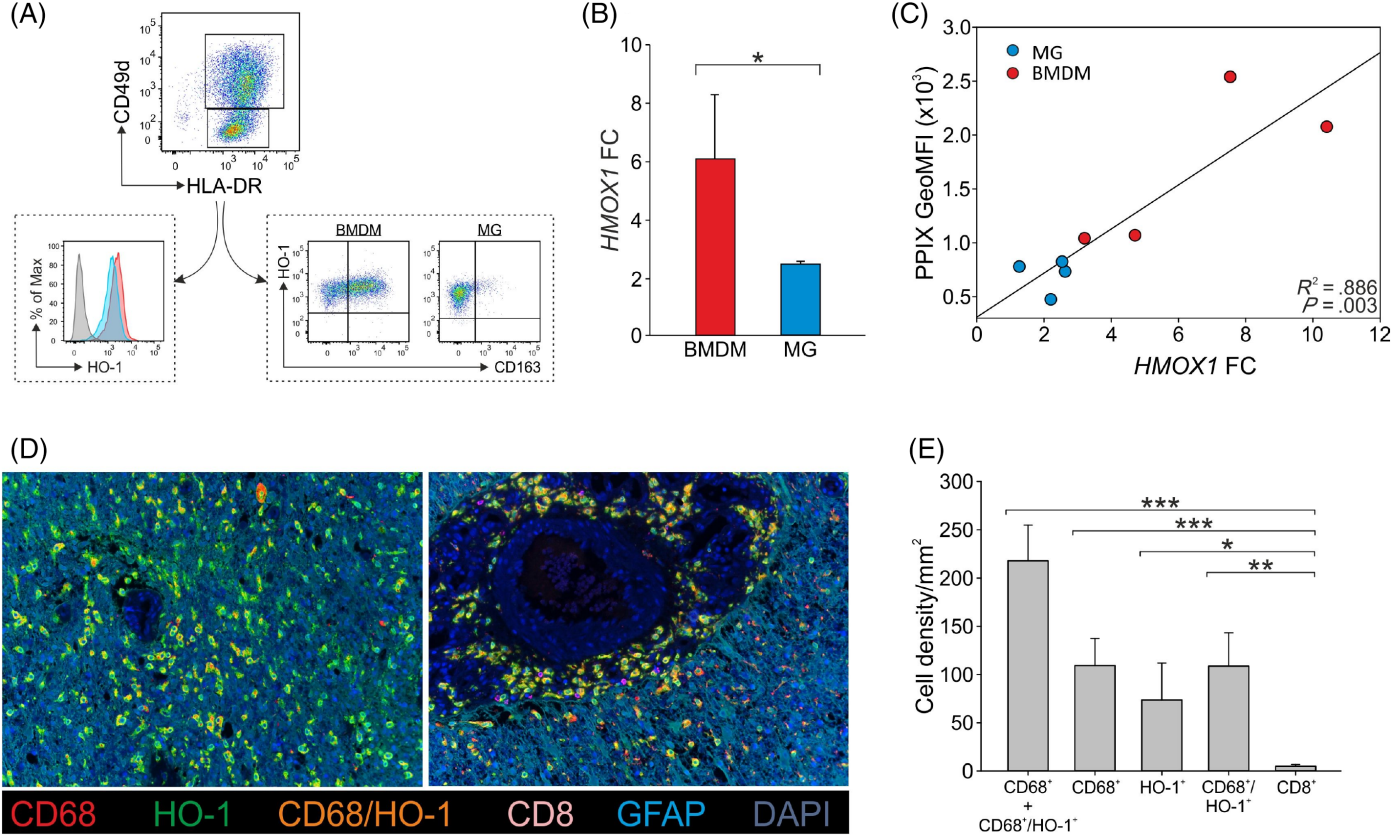
HO‐1 expression in BMDMs and MG in GBM tissue. (A) Representative flow cytometry analysis of HO‐1 and CD163 protein expression in BMDMs and MG. After dead cell exclusion and determination of CD45^+^ cells, BMDMs and MG cells were further identified in the CD33^+^ gate as HLA‐DR^+^/CD49d^+^ and HLA‐DR^+^/CD49d^−^, respectively (upper part of the figure). In the bottom part, on the left, the red peak represents the HO‐1 mean fluorescence intensity (MFI) of BMDMs, while the light blue peak corresponds to the HO‐1 MFI of MG cells. HO‐1^+^ cells were defined using fluorescence minus one (FMO) control (gray peak). On the right part of the figure, HO‐1/CD163 co‐expression is shown in BMDMs and MG cells. (B) *HMOX1* gene expression level analyzed by means of qRT‐PCR in BMDMs (red bar) and MG (blue bar), sorted from GBM specimens by a FACS sorter and expressed as a fold change (FC). *HMOX1* expression was normalized to the *β‐actin* gene. Mean ± SE of four GBM patients. Comparison by Mann‐Whitney test. *<.05. (C) Correlation between PPIX fluorescence emission and *HMOX1* FC in BMDMs (red dots) and MG (blue dots). Pearson correlation on four paired samples. (D) Representative composite images showing two fields of the same GBM tissue labeled with CD68 (red, macrophages), HO‐1 (green), CD8 (pink, T cells) and GFAP (light blue, tumor cells); nuclei were counterstained with DAPI (blue). Magnification ×20. (E) Analysis of the immune infiltrate by multispectral imaging. The bar plots show the mean ± SE of cell density per megapixel of CD68^+^ and CD68^+^/HO‐1^+^ cells together, CD68^+^, HO‐1^+^, CD68^+^/HO‐1^+^ and CD8^+^ cells calculated by considering 10 different fields from the FFPE tissue slides of nine GBM patients. Comparison by Mann‐Whitney test. *<.05; **<.01; ***<.001

Our previous study disclosed a sustained iron‐recycling metabolism in immune suppressive BMDMs present in high‐grade gliomas, with a significant overexpression of several genes implicated in iron metabolism.^11^ Herein, we specifically focused on *HMOX1*, the gene coding for HO‐1, which was significantly overexpressed in BMDMs compared to resident MG and tumor cells.^11^ HO‐1 protein staining on cell suspensions from freshly resected GBM specimens confirmed that both macrophage populations stained positively, with a higher intensity in BMDMs compared to MG cells (Figure 1A). We previously showed that CD163, that is the scavenger receptor for the hemoglobin‐haptoglobin complex, is upregulated in BMDMs infiltrating GBM tissues,^11^ and thus we evaluated its expression on GBM cell suspensions at protein level and observed a high proportion of BMDMs co‐expressing CD163 and HO‐1, as opposed to MG cells (Figure 1A), thus reinforcing the notion that BMDMs have a pronounced iron metabolism in the TME of GBM.

We then sorted CD45^+^/HLA‐DR^+^/CD49d^+^ cells (BMDMs) and CD45^+^/HLA‐DR^+^/CD49d^−^ cells (MG) from the central intense fluorescence layer of GBM specimens based on 5‐ALA‐assisted surgery and confirmed the higher *HMOX1* expression in BMDMs compared to MG by qRT‐PCR (Figure 1B, *P* = .029). Furthermore, a significant positive correlation was observed between PPIX fluorescence emission and *HMOX1* expression in BMDMs and MG cells (Figure 1C, *R* ^2^ = .886, *P* = .00342). PPIX fluorescence emission by tumor‐infiltrating macrophages can thus be used as an indicator of iron metabolism in the two different macrophage subsets.

In order to visualize the presence of HO‐1^+^ cells in GBM tissues, we stained FFPE tissue slides by multispectral imaging and evaluated the expression of this marker together with CD8, CD68 and GFAP. This evaluation confirmed a large presence of CD68^+^/HO‐1^+^ macrophages (Figure 1D, orange cells), which mainly had two patterns of expression: in the areas surrounding the necrotic tissue or spread in the tumor tissue. Furthermore, a quantitative analysis of the immune cell composition indicated that HO‐1‐expressing macrophages (Figure 1E, CD68^+^/HO‐1^+^ mean of cell density = 108.68 ± 34.65) represented about half of the total macrophages (Figure 1E, CD68^+^ and CD68^+^/HO‐1^+^ cells mean = 217.91 ± 36.98). As expected, CD8^+^ T lymphocytes were present at much lower percentages (Figure 1E, CD8^+^ mean = 4.87 ± 1.85), in line with our previous data.^11^

### 3.2 HO‐1 inhibition restores T cell proliferation suppressed by macrophages

**FIGURE 2:**
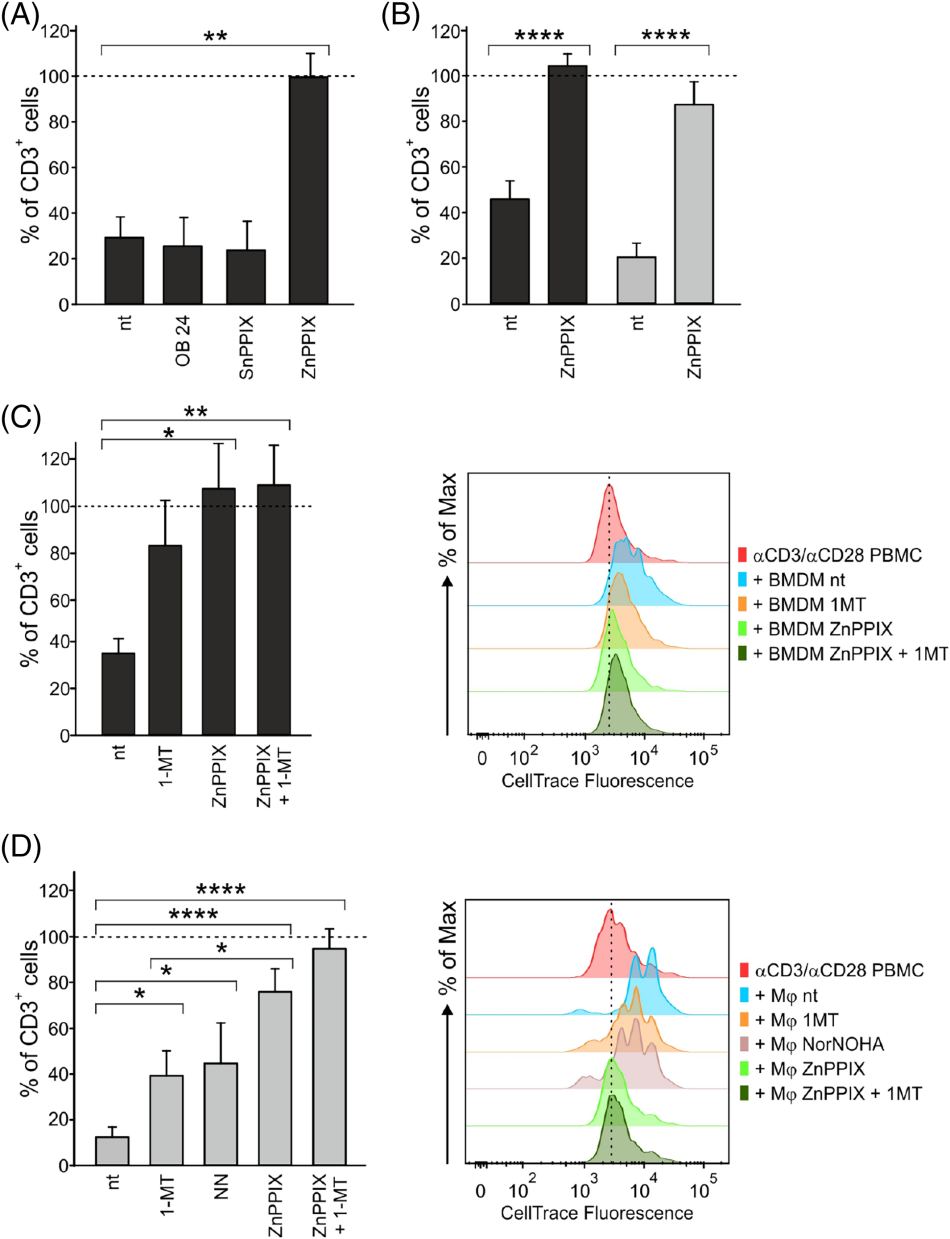
HO‐1 inhibition in myeloid cells relieves immune suppression exerted on T cells. T cells were cultured with BMDMs (black bars) isolated from GBM specimens or with Mφ (gray bars) for 96 hours; data were normalized assuming the proliferation of T cells alone to be 100% (represented by a dotted line). The bars represent the mean ± SE. Comparison by *t*‐test. *<.05; **<.01; ****<.0001. (A) Proliferation of T cells cultured with BMDMs (ratio 1:1) isolated by immunomagnetic sorting from the central region of GBM specimens, untreated (nt) or pretreated with HO‐1 inhibitors (n = 3). (B) Proliferation of T cells cultured with BMDMs (black bars, ratio 1:1) isolated by immunomagnetic sorting from the central region of GBM specimens (n = 7) or with Mφ (gray bars, ratio 1:0.5, n = 10), nt or pretreated with 5 μM ZnPPIX. (C,D) Proliferation of T cells in the presence of (C) BMDMs and (D) Mφ, nt, pretreated with 5 μM ZnPPIX or treated in co‐culture with 1 mM 1‐MT or 0.5 mM NN (C: n = 4, except for ZnPPIX + 1‐MT—n = 3; D: nt and 1‐MT—n = 9, ZnPPIX—n = 8, NN—n = 5, ZnPPIX + 1‐MT—n = 6). On the right side of each panel, CellTrace histograms from two representative experiments with either BMDMs or Mφ. αCD3/αCD28 PBMCs (red plots, with dashed line) are set as a control of proliferation

As BMDMs are the prevalent immune cells in the TME of GBM and are endowed with the highest iron metabolism and immune suppressive ability,^11^ we hypothesized an association between the two pathways. In order to show that HO‐1 drives immune suppression not only in murine macrophages^13^, ^14^, ^15^, ^16^ but also in human BMDMs, we used a pharmacological approach to inhibit HO‐1 enzymatic activity. BMDMs were thus isolated from the central area of GBM lesions, pretreated with different HO‐1 inhibitors and then tested for their ability to suppress the proliferation of activated T cells. Since HO‐1 inhibitors could have a direct effect on T cells,^19^ BMDMs were pretreated with the selected drugs and then washed to completely remove them before the co‐culture. Pretreatment with one of these inhibitors, ZnPPIX, enabled the complete recovery of T cell proliferation, while the other inhibitors did not alter it (Figure 2A). Accordingly, we strengthened this finding in another group of experiments with BMDMs separated from GBM samples (Figure 2B) and confirmed it in an in vitro model of immunosuppressive macrophages derived from blood monocytes of HDs (Figure 2B, Mφ), whose relevant suppressive activity and HO‐1 expression (Figure S1) justified their use in investigating HO‐1‐related immunosuppressive mechanisms. Previous studies from us and others identified the contribution of the IDO1 and ARG‐1 enzymes in different immunosuppressive myeloid populations.^7^, ^20^, ^21^ For this reason, we evaluated the effect of the IDO1 inhibitor 1‐MT and ARG‐1/ARG‐2 inhibitor Nor‐NOHA (NN). Results with sorted BMDMs revealed a partial recovery of T cell proliferation following the addition of 1‐MT and a complete rescue of T cell proliferation with ZnPPIX (Figure 2C). When Mφ were tested, IDO1 and ARG‐1/ARG‐2 inhibitors significantly, but not completely, restored T cell proliferation (Figure 2D) and an even higher recovery was observed with ZnPPIX treatment.

### 3.3 Blocking of HO‐1 activity alters the expression of molecules involved in the immunosuppressive pathways

**FIGURE 3:**
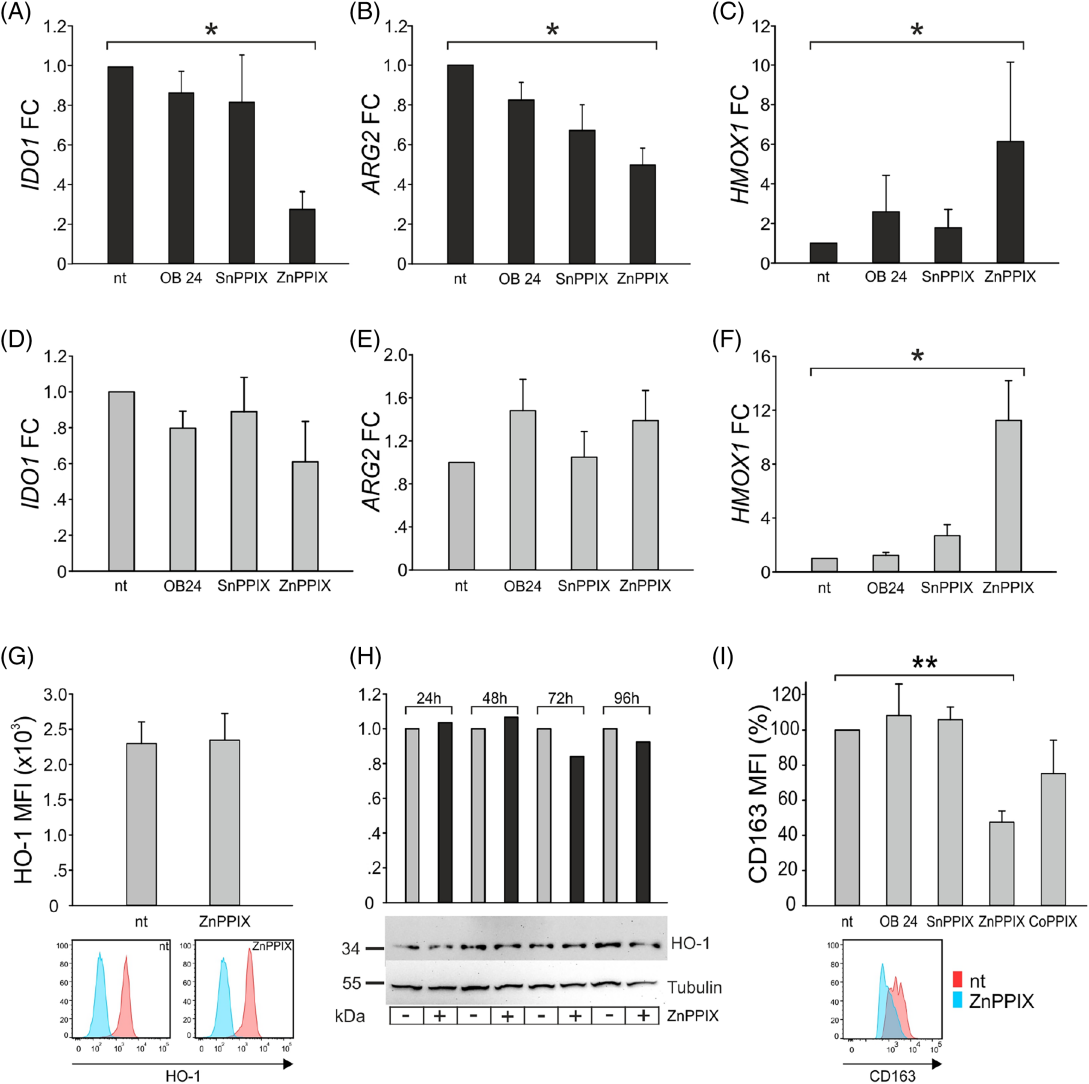
Modulation of molecules involved in immunosuppression after HO‐1 inhibition. Expression (FC) of (A) *IDO1*, (B) *ARG2* and (C) *HMOX1* by qRT‐PCR in BMDMs (black bars) from GBM specimens 24 hours after treatment with HO‐1 inhibitors. Mean ± SE of four experiments. Comparison by Mann‐Whitney test. *<.05. Expression of (D) *IDO1*, (E) *ARG2* and (F) *HMOX1* by qRT‐PCR in Mφ (gray bars) from HDs. Mean ± SE of four experiments. Comparison by Mann‐Whitney test. *<.05. (G) HO‐1 protein evaluation by flow cytometry in Mφ, nt or treated with 5 μM ZnPPIX. On the upper part, the bars represent the average MFI of HO‐1 protein ± SE of three experiments. On the bottom, representative flow cytometry plots of HO‐1 expression in nt (left) and ZnPPIX‐treated (right) Mφ. For each plot, the light blue peak corresponds to cell autofluorescence. (H) Western blot analysis of HO‐1 protein in Mφ, untreated (gray histograms) or treated with 5 μM ZnPPIX (black histograms). Cellular fractions were collected every 24 hours from the treatment for 4 days and hybridized with anti‐HO‐1 and anti‐α‐tubulin antibodies. The bars represent the HO‐1 expression normalized to each time point's untreated condition and calculated relative to the loading control. (I) CD163 expression in Mφ treated with HO‐1 modulators. On the upper part, the bars represent the average MFI calculated as a percentage reduction of CD163 in treated cells compared to untreated samples, set at 100%. The whiskers show the SE (n = 3 for OB 24 and SnPPIX; n = 5 for nt and ZnPPIX; n = 4 for CoPPIX). Comparison by Mann‐Whitney test. **<.01. On the bottom, representative flow cytometry plots of CD163 expression in nt (red histogram) and ZnPPIX‐treated (light‐blue histogram) Mφ

To understand the downstream effects of HO‐1 inhibition, we analyzed *HMOX1* expression after treatment with the different HO‐1 inhibitors. The expression of other genes involved in the immunosuppressive features of myeloid cells, such as *IDO1*, *ARG1* and *ARG2*, was also assessed. While *ARG1* was not expressed at a detectable level in BMDMs and in other brain tissue cells, the expression of *ARG2* was present both in macrophages and tumor cells (data not shown). Although the most common observation is the expansion and accumulation of ARG1‐producing tumor‐infiltrating myeloid cells,^22^ local immune suppression can also be driven by ARG‐2.^23^, ^24^ ZnPPIX treatment significantly reduced the expression of *IDO1* (Figure 3A) and *ARG2* (Figure 3B) in BMDMs, while inducing high *HMOX1* expression (Figure 3C). This paradoxical effect is in line with previous studies^25^, ^26^ and it has been hypothesized that ZnPPIX, acting as a heme analogue, removes the transcriptional Bach1 repressor, inducing *HMOX1* transcription through Nrf2 activation.^27^ Similar results were obtained in in vitro‐derived Mφ in which ZnPPIX significantly induced *HMOX1* (Figure 3F) but did not significantly reduce *IDO1* (Figure 3D) and *ARG2* expression (Figure 3E). Interestingly, pretreatment of Mφ with the HO‐1 inducer CoPPIX increased *IDO1*, *ARG2* and *HMOX1* expression (data not shown). These results prompted us to evaluate HO‐1 expression at the protein level by means of flow cytometry (Figure 3G) and immunoblotting (Figure 3H) in ZnPPIX‐treated Mφ. The results indicated that the HO‐1 protein level in treated macrophages was comparable to that of untreated cells (Figure 3G,H) and did not change after 4 days (Figure 3H), thus demonstrating that HO‐1 protein synthesis is not increased after the upregulation of its transcript.

Besides the previously mentioned molecules, we also analyzed CD163, in order to understand whether HO‐1 inhibition also influenced its expression. We measured its protein level by flow cytometry after HO‐1 modulation and observed its significant decrease after 24 hours from ZnPPIX treatment (Figure 3I), reinforcing the connection between this marker and HO‐1 in the iron pathway.

### 3.4 Upregulation of PD‐L1 expression is mediated by STAT3 and depends on the crosstalk with activated T cells

**FIGURE 4:**
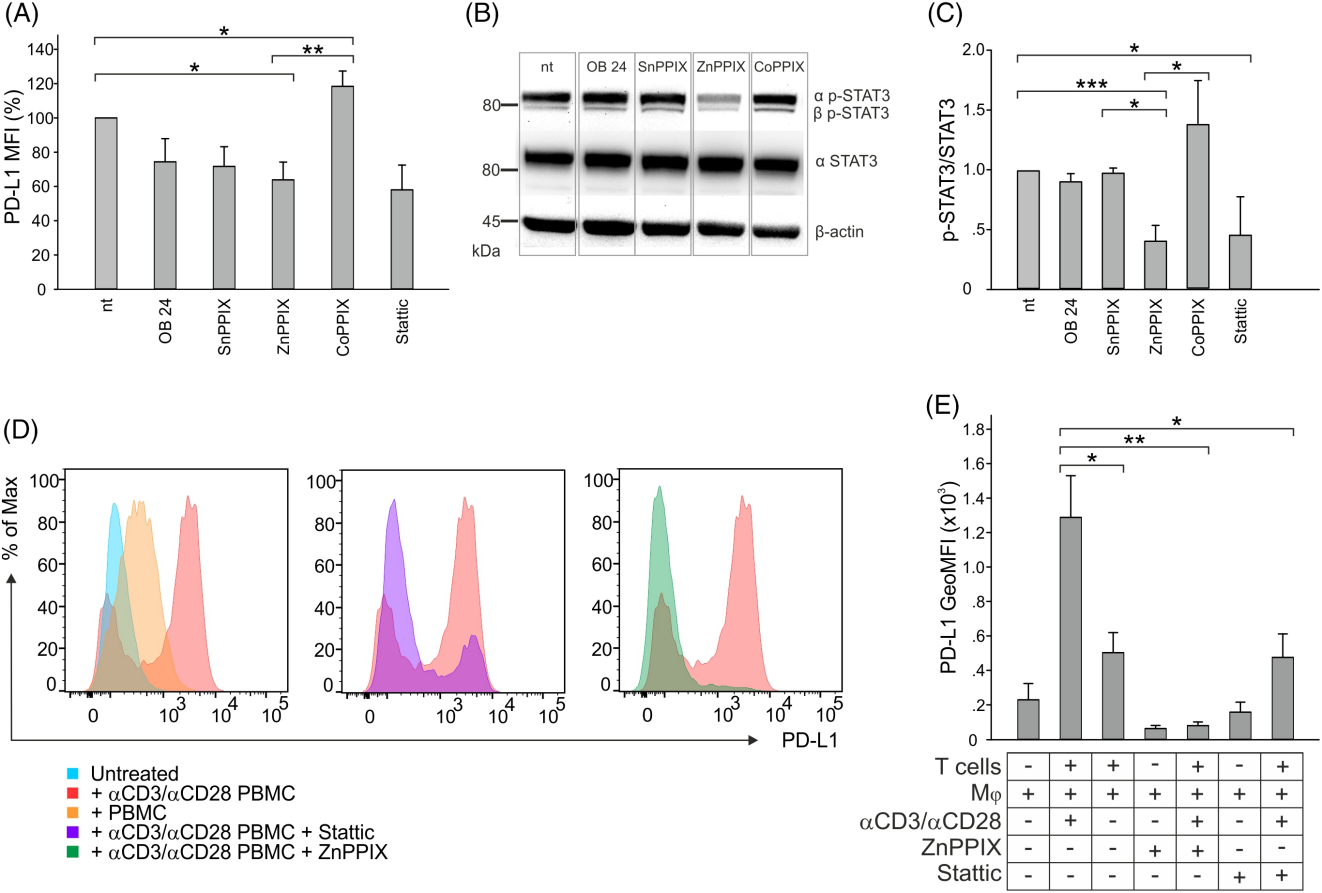
Modulation of PD‐L1 by HO‐1. (A) PD‐L1 expression in Mφ treated with HO‐1 modulators. The bars represent the average MFI calculated as a percentage reduction of PD‐L1 in treated cells compared to untreated cells, set at 100%; the whiskers denote the SE (n = 4, except for Stattic n = 3). Comparison by *t*‐test. *<.05; **<.01. (B) Western blot analysis of p‐STAT3 (MW = 86 kDa) and STAT3 (MW = 79 kDa) proteins in Mφ, untreated or treated with HO‐1 modulators. β‐Actin (MW = 42 kDa) was used as a control. (C) The bars represent the average p‐STAT3/STAT3 protein expression ratio normalized to the nt condition ± SE (n = 3, except for CoPPIX—n = 4 and ZnPPIX—n = 5). Comparison by *t*‐test. *<.05; ***<.001. (D) Representative flow cytometry histograms of PD‐L1 expression in Mφ cultured with or without activated or resting T cells for 24 hours. (E) The bars represent the average geometric MFI of PD‐L1 ± SE (n = 3). Comparison by *t*‐test. *<.05; **<.01

To further understand the mechanisms involved in HO‐1‐mediated immunosuppression, we evaluated by flow cytometry whether the different HO‐1 inhibitors or the inducer CoPPIX were able to modulate the surface expression of PD‐L1, whose binding to PD‐1 on the T cell surface reduces immune responses and maintains immune tolerance.^28^ Since PD‐L1 expression is controlled by the activation of STAT3,^29^ we also evaluated the latter by Western blot. We found a significant downregulation of PD‐L1 in in vitro‐derived Mφ following ZnPPIX treatment and a similar trend was also observed with the HO‐1 inhibitors OB 24 and SnPPIX (Figure 4A). Mφ showed a reduction in PD‐L1 expression (Figure 4A, *P* = .057) and in STAT3 activation (Figure 4C, *P* = .022) after treatment with the STAT3 inhibitor Stattic, and a similar downregulation of STAT3 activation was observed when HO‐1 was inhibited with ZnPPIX (Figure 4B,C). Conversely, treatment with the HO‐1 inducer CoPPIX increased both PD‐L1 expression (Figure 4A) and STAT3 activation (Figure 4B,C). Taken together, these results indicate that PD‐L1 regulation depends on HO‐1 activity.

We previously demonstrated that the suppressive activity of myeloid‐derived suppressor cells (MDSCs) is influenced by the T cell activation status, since the proliferation, differentiation and immunosuppressive activity of MDSCs are fueled by the contact with activated, but not with resting, T cells.^20^ In addition, we demonstrated a strong induction of PD‐L1 after contact with activated T cells, mediated by STAT3.^20^ Therefore, we then evaluated the modulation of PD‐L1 expression on suppressive Mφ following contact with T cells and, in line with our previous results, we observed that activated (Figure 4E, treated with αCD3/αCD28), but not resting (Figure 4E, without αCD3/αCD28), T cells induced a strong upregulation of PD‐L1 (Figure 4D,E). Worth noting is that this effect was significantly abrogated by ZnPPIX and Stattic treatment (Figure 4D,E).

Taken together, these results reinforce the previous findings, that is, that the mechanism of immune suppression exerted by myeloid cells depends on the contact with activated T cells, and additionally stress the role of HO‐1 in controlling PD‐L1 expression through STAT3 phosphorylation.

### 3.5 Myeloid HO‐1 affects IDO1 activity

**FIGURE 5:**
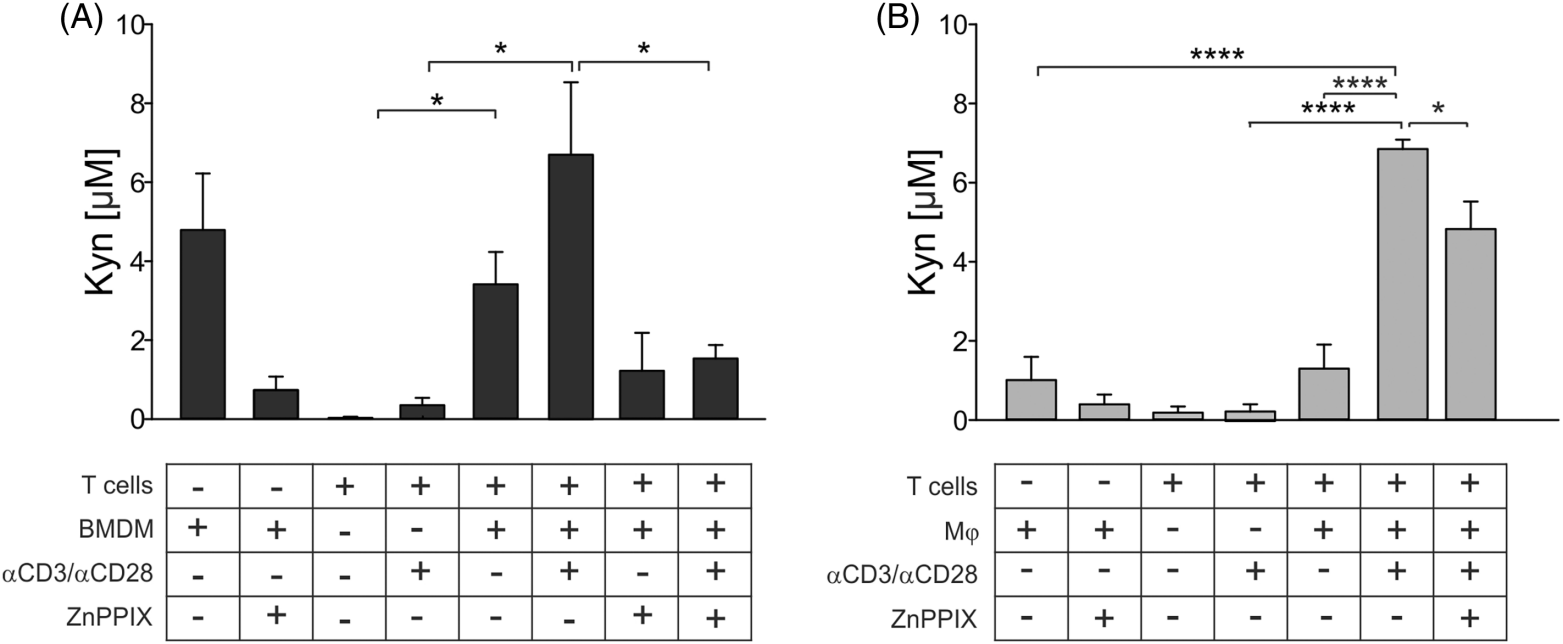
IDO1 activity measured by HPLC as L‐kynurenine release in cell supernatants. (A) BMDMs isolated from GBM specimens and (B) Mφ, untreated or treated with 5 μM ZnPPIX, were cultured for 24 hours with activated or resting T cells. The bars represent the median ± SE (A: n = 5 except for T‐cells + BMDM ZnPPIX, BMDM alone, BMDM ZnPPIX, αCD3/αCD28‐T‐cells and T‐cells alone n = 3; B: n = 7, except for αCD3/αCD28‐T‐cells and T‐cells alone n = 5). Comparison by *t*‐test. *<.05; ****<.0001

To evaluate the relationship between IDO1 and HO‐1, we measured IDO1 enzymatic activity in terms of Kyn production in culture supernatants of suppressive myeloid cells with resting or activated T cells and following HO‐1 inhibition. Interestingly, BMDMs had a spontaneous and sustained Kyn release, which had a trend toward an increase in the presence of activated T cells. However, blocking of HO‐1 activity with ZnPPIX significantly reduced their Kyn production (Figure 5A, *P* = .0242). Analogously, the block of HO‐1 activity also reduced IDO1 activity when Mφ were cultured with activated T cells (Figure 5B, *P* = .0175). Activated T cells hardly produced Kyn, indicating that Kyn production mainly depends on myeloid cells, following contact with T cells.

All together, these results highlight the importance of HO‐1 in controlling IDO1 activity in myeloid cells.

### 3.6 Myeloid HO‐1 modulates IL‐10 release in the crosstalk between macrophages and T cells

Several lines of evidence demonstrated a link between the HO‐1 pathway and IL‐10 signaling, generating a positive feedback that amplifies macrophage antiinflammatory and immune suppressive responses.^30^, ^31^, ^32^ To verify whether IL‐10 release is implicated in the myeloid HO‐1 suppressive pathway, we cultured Mφ, with or without HO‐1 inhibition, with resting or activated T cells and IL‐10‐expressing Mφ and T cells were then evaluated.

**FIGURE 6:**
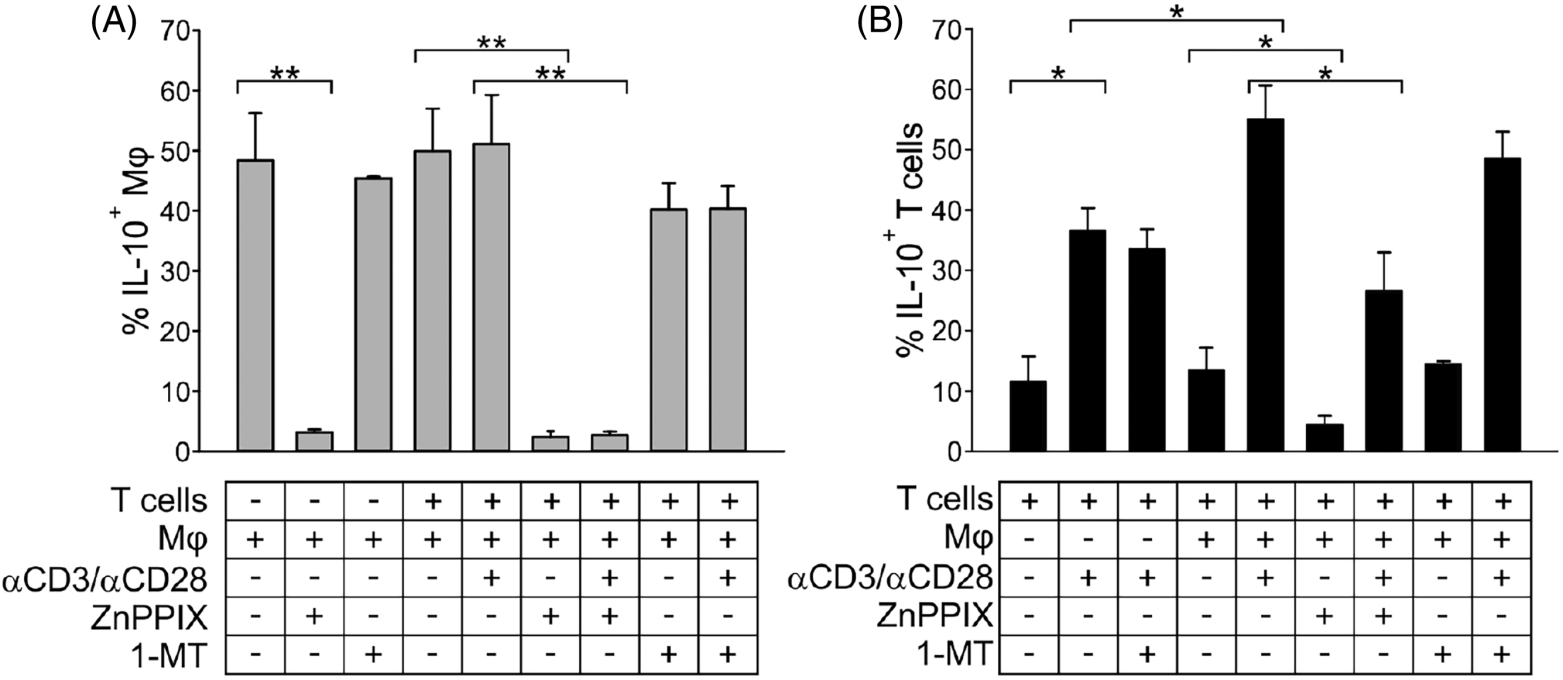
The role of HO‐1 in IL‐10 release by Mφ and T cells upon contact. Mφ, untreated, pre‐treated with 5 μM ZnPPIX or treated in co‐culture with 1 mM 1‐MT, were cultured with or without T cells for 14 hours. IL‐10‐expressing cells were evaluated. Comparison by Mann‐Whitney test. *<.05; **<.01. The bars represent the mean ± SE of (A) IL‐10‐expressing Mφ and (B) IL‐10‐expressing T cells (n = 3 for αCD3/αCD28‐T‐cells + 1‐MT; n = 4 for T cells, T‐cells + Mφ 1‐MT, αCD3/αCD28‐T‐cells + Mφ 1‐MT, Mφ 1‐MT; n = 5 for T‐cells + ZnPPIX‐treated Mφ, αCD3/αCD28‐T‐cells + ZnPPIX‐treated Mφ, ZnPPIX‐treated Mφ; n = 6 for αCD3/αCD28‐T‐cells, T‐cells + nt Mφ, αCD3/αCD28‐T‐cells + nt Mφ, nt Mφ)

A high percentage of Mφ had a basal expression of IL‐10, which was unaffected by the contact with T cells; however, HO‐1 inhibition significantly reduced this percentage (Figure 6A, *P* = .004); conversely, no significant reduction was observed after IDO1 inhibition with 1‐MT (Figure 6A), thus excluding the connection of IL‐10 with the IDO1 immunosuppressive pathway. Regarding T cells, the activation with anti‐CD3 and anti‐CD28 mAbs significantly upregulated the frequency of cells expressing IL‐10 compared to the condition with resting T cells (Figure 6B, *P* = .010) in line with our previous data^20^ and such percentage was further increased upon contact with immunosuppressive Mφ (Figure 6B, *P* = .026). Interestingly, a significant reduction in the percentage of IL‐10‐expressing T cells after contact with ZnPPIX‐treated Mφ was also observed (Figure 6B, *P* = .017), while blocking IDO1 did not alter it.

It thus appears that HO‐1 plays a key role in IL‐10 release, which is involved in the interplay between myeloid and T cells, and its inhibition in Mφ influences in turn the IL‐10 release by T cells in contact with them.

## 4 DISCUSSION

The development of novel and more effective therapeutic approaches for GBM patients is of paramount importance. Its genetic heterogeneity alone cannot explain the lack of efficacy of standard therapies, and the identification of an immunosuppressed TME, mainly established by TAMs and by the phenomenon of T cell exhaustion, substantiates the need for combinatorial approaches.^33^, ^34^ Our study examined the role of a key enzyme in the iron metabolism of immunosuppressive macrophages largely present in the microenvironment of human GBM. We found that immune suppression and iron metabolism are inextricably linked in such cells, and that blocking HO‐1 alleviates the immune suppression they exert. Given the relevance of blood‐derived macrophages in establishing an immune suppressed microenvironment in GBM, finding an appropriate target to block their tolerogenic activity has clinical relevance.

HO‐1 overexpression is commonly present in human cancers, including gliomas, and its expression level is positively correlated with disease stage and poor prognosis of patients.^35^, ^36^, ^37^, ^38^ In addition to its expression in tumor cells, our group and others have documented the presence of HO‐1 in the immune microenvironment, particularly in cancer tissue TAMs.^11^, ^14^, ^15^, ^17^, ^39^ In particular, its role in orchestrating the immunosuppressive activity of TAMs was recently documented in mouse models,^13^, ^14^, ^15^ together with its function in the formation of metastases,^17^ and several of these studies in preclinical models clearly demonstrate that HO‐1 inhibition can be exploited as a novel therapeutic strategy for treating different clinical conditions. A number of synthetic metalloporphyrins have been initially studied in vitro and in vivo for their clinical efficacy for the treatment of hyperbilirubinemia, but more recently they have shown a therapeutic potential also for the treatment of cancer. Preclinical studies in mouse models showed that targeting HO‐1 by the inhibitor SnPPIX resulted in a therapeutic effect^15^ and synergized with chemotherapy,^14^ while HO‐1 inhibition with another inhibitor, OB 24, overcame resistance to immunotherapy in a mouse melanoma model and was used to improve checkpoint inhibitors therapy.^16^

However, given the presence of two HO isoforms, an inducible form (ie, HO‐1) and a constitutive one (ie, HO‐2), it should be considered that each compound has a different inhibitory potency toward the two isoforms. For example, SnPPIX is the most specific in inhibiting HO‐2 over HO‐1, while ZnPPIX is known to be the least inhibitory toward HO‐2.^40^

In this work, we extend the previous findings and show that HO‐1 inhibition abrogates immune suppression exerted by BMDMs separated from human GBM tissue specimens (Figure 2) and we demonstrate that, among the different HO‐1 inhibitors used, ZnPPIX is the most effective in modulating their activity.

We show that the blockage of myeloid HO‐1 activity reduces significantly *IDO1* gene expression (Figure 3) and activity (Figure 5). In this regard, it has been recently demonstrated that Kyn production by IDO1‐expressing cells suppresses ferroptotic cell death, thus suggesting that IDO1‐expressing cells in the TME may assist tumor progression also through the activation of a cell survival pathway.^41^ Our results obtained with BMDMs isolated from GBM patients (Figure 5A) are in line with this hypothesis, since BMDMs have a sustained spontaneous production of Kyn. Of note, Kyn generation is significantly reduced in the presence of ZnPPIX, strongly suggesting a link between HO‐1 and IDO1 metabolism.

The concomitant reduction in PD‐L1 expression (Figure 4) and IL‐10 release (Figure 6), observed after ZnPPIX treatment, suggests that this treatment affects immune suppressive mechanisms that occur through both cell‐to‐cell contact and soluble factors, which are induced in the presence of activated, but not resting, T cells. The reduction in IL‐10 production observed in both T cells and macrophages (Figure 6) is in line with other studies describing the release of IL‐10 downstream of HO‐1.^42^, ^43^ Furthermore, we show that HO‐1 controls PD‐L1 expression through the phosphorylation of STAT3 (Figure 4B,C), which is also involved in IL‐10 transcription^44^, ^45^ and in several immune suppressive mechanisms.^46^ Taken together, these findings suggest that HO‐1 may be a target to relieve immune suppression in the GBM microenvironment, providing a promising translational approach.

The local delivery of drugs has already been explored in glioma management^47^ and our group has demonstrated a selective phagocytosis of lipid nanocapsules (LNCs) in both circulating and tumor‐associated myeloid cells from GBM patients.^48^ ZnPPIX‐loaded nanoparticles could thus be proposed as a strategy to overcome GBM multifactorial immunosuppression and to increase antitumor immune responses by reprogramming immunosuppressive myeloid cells. In this regard, HO‐1 inhibition with ZnPPIX in Mφ also resulted in the reduction of the CD163 protein level (Figure 3I), a common marker of tumor‐promoting and antiinflammatory macrophages, whose expression by TAMs and cancer cells has been shown to be a strong indicator of poor prognosis in several human cancers, including gliomas.^49^, ^50^

Overall, these results underline a new aspect of the activity of tumor‐promoting macrophages in the TME of GBM, in which their immunoregulatory ability is entangled with a metabolic pathway. By targeting iron metabolism through the inhibition of HO‐1, our study demonstrates the feasibility of hitting key immune suppressive mechanisms while simultaneously reprogramming protumoral macrophages, representing a new therapeutic strategy for future clinical studies in combination with immune stimulation.

## AUTHOR CONTRIBUTIONS

**Sara Magri:** Conceptualization, Methodology, Formal analysis and investigation, Writing ‐ original draft preparation, Writing ‐ review and editing. **Beatrice Musca:** Formal analysis and investigation, Writing ‐ original draft preparation, Writing ‐ review and editing. **Laura Pinton:** Formal analysis and investigation. **Elena Orecchini:** Formal analysis and investigation. **Laura Belladonna:** Formal analysis and investigation, Resources. **Ciriana Orabona:** Formal analysis and investigation, Resources. **Camilla Bonaudo:** Resources. **Francesco Volpin:** Resources. **Pietro Ciccarino:** Resources. **Valentina Baro:** Resources. **Alessandro Della Puppa:** Resources. **Susanna Mandruzzato:** Conceptualization, Formal analysis and investigation, Writing ‐ original draft preparation, Writing ‐ review and editing, Funding acquisition, Supervision. The work reported in the article has been performed by the authors, unless clearly specified in the text.

## FUNDING INFORMATION

This work was supported by TRANSCAN‐2, ERA‐NET to S.M., Università degli Studi di Padova (BIRD205873 to S.M.), Ministero della Salute (RF‐2019‐12369251 to S.M.) and Intramural Research Funding Programs IOV‐IRCCS (BIOV19MANDR to S.M.).

## CONFLICT OF INTEREST

The authors report that there are no competing interests to declare.

## ETHICS STATEMENT

Our study was conducted in accordance with the principles of the Declaration of Helsinki. All the experiments were approved by the ethical committees of the Veneto Institute of Oncology IRCCS of Padova, Italy (MDSC_SNC 2016/13), and the Padova and Florence University Hospitals (NOI_NCH 1536/19). All participants included in our study gave their written informed consent.

## Supporting information


**Appendix S1** Supporting Information.

## Data Availability

The data supporting the findings of our study are available in the Zenodo repository at 10.5281/zenodo.6510838, upon reasonable request from the corresponding author, Susanna Mandruzzato.
