# Supplementary material for: The immunosuppression pathway of tumor‐associated macrophages is controlled by heme oxygenase‐1 in glioblastoma patients
Source: Int J Cancer. 2022 Sep 15;151(12):2265–77. doi: 10.1002/ijc.34270 (PMC9825884; doi:10.1002/ijc.34270)
Supplement: Supplementary file 1 — Appendix S1 Supporting Information. [file IJC-151-2265-s001.pdf]

The immunosuppression pathway of tumor-associated macrophages is controlled by heme oxygenase-1 in glioblastoma patients

Sara Magri, Beatrice Musca, Laura Pinton, Elena Orecchini, Laura Belladonna, Ciriana Orabona, Camilla Bonaudo, Francesco Volpin, Pietro Ciccarino, Valentina Baro, Alessandro Della Puppa, Susanna Mandruzzato

Table of contents:

Supplementary Methods

Supplementary Figure

Supplementary Methods

In vitro differentiation of immunosuppressive macrophages

Peripheral blood mononuclear cells (PBMCs) were isolated from buffy coats from healthy donors (HDs). After 1:3 dilution in DPBS (Dulbecco's Phosphate Buffer Saline), PBMCs were separated by density gradient centrifugation on Ficoll-Paque PLUS (GE Healthcare-Amersham, Buckinghamshire, UK), washed with PBS implemented with 1% human serum (HS) and resuspended in IMDM culture medium (Iscove's Modified Dulbecco's Medium, Life Technologies, Thermo Fisher Scientific, Waltham, MA, USA) supplemented with 10% FBS, 10 mM Hepes Buffer, 100 U/mL penicillin/streptomycin, 0.28 mM asparagine, 1.5 mM glutamine, 0.67 mM arginine and 20 μM β-mercaptoethanol for cell count. The percentage of monocytes in the PBMC fraction was determined through scatter parameters using a BD™ LSRII flow cytometer (BD Biosciences, Becton Dickinson, Franklin Lakes, NJ, USA). PBMCs containing $3x10^5$ monocytes were seeded in each well of 24-well plates. To favor monocyte adhesion, cells were resuspended in a low serum-content RPMI 1640 medium supplemented with 3% HS, 10 mM Hepes Buffer, 100 U/mL penicillin/streptomycin, 0.28 mM asparagine, 1.5 mM glutamine and 0.67 mM arginine. After 1 hour at 37°C, 5% CO_2, the adherent monolayer of monocytes was gently washed with no supplemented RPMI medium to remove lymphocytes and platelets. Monocytes were differentiated for seven days in RPMI 1640 medium supplemented with 10% FBS, 10 mM Hepes Buffer, 100 U/mL penicillin/streptomycin, 0.28 mM asparagine, 1.5 mM glutamine, 0.67 mM arginine, and 100 ng/mL macrophage colony-stimulating factor (M-CSF, Miltenyi Biotec, Bergisch Gladbach, Germany). After four days, one-third of the medium volume was removed and replaced with fresh complete medium containing 100 ng/mL M-CSF.

Evaluation of the immunosuppressive activity of macrophages

To evaluate the immunosuppressive activity of both BMDMs isolated from GBM specimens and *in vitro*-derived Mφ, PBMCs from HD buffy coats were stained with 0.5 μM CellTrace™ Violet Cell

Proliferation Kit (Invitrogen, Molecular Probes, MA, USA), according to the manufacturer's instructions. CellTrace™-labeled PBMCs were activated with coated 1 µg/mL anti-CD3 and 5 µg/mL soluble anti-CD28 (BioLegend, San Diego, CA, USA) and co-cultured in flat bottom 96- or 384-well plates at 1:1 or 1:0.5 ratios with myeloid cells for four days at 37°C, 5% CO_2, in arginine-free RPMI supplemented with 150 µM arginine, 10% FBS, 10 U/mL penicillin/streptomycin and 10 mM Hepes Buffer. On the fourth day, the cells were collected and stained with anti-CD3 PE-Cy7 (Beckman Coulter, California, USA) for flow cytometry analysis using the BD™ LSRII flow cytometer (BD Biosciences). T cell proliferation in the different conditions was evaluated by calculating the absolute number of CellTrace™-labeled CD3-positive T cells using TruCount™ tubes (BD Biosciences), containing fluorescent beads that permit a quantitative measurement of cells. Data were normalized by assuming the proliferation of T cells cultured alone as 100%.

Supplementary Figure

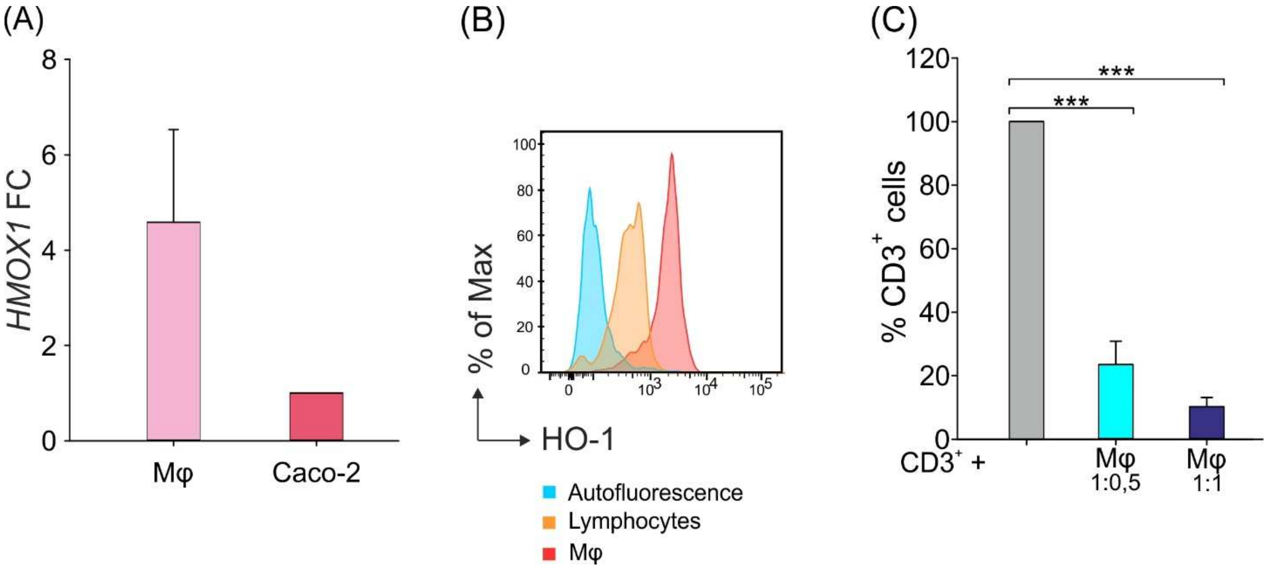

Supplementary Fig. 1. Heme oxygenase-1 expression and immune suppressive activity of *in vitro*-derived macrophages. **(A)** *HMOX1* gene expression level was analyzed using qRT-PCR from macrophages (Mφ) differentiated *in vitro* from HD monocytes. *HMOX1* expression was normalized to *β-ACTIN* as a housekeeping gene and expressed as a fold change (FC) compared to the reference cell line Caco-2. Histograms show the mean ± SE of seven HDs. **(B)** Representative flow cytometry plots of HO-1 fluorescence intensity evaluation in Mφ (red histogram) compared to lymphocytes (orange histogram) and autofluorescence (blue histogram). The average MFI (mean fluorescence intensity) of HO-1 protein in Mφ evaluated by intracytoplasmic staining is 2250±227.5 (n=4). **(C)** Percentages of proliferation of allogeneic T cells activated with αCD3/αCD28 mAbs and cultured with Mφ at a ratio of 1:0.5 (light blue bar) and 1:1 (blue bar) compared to activated T cells cultured alone (grey bar) (n=8). Comparison by Mann-Whitney test. *** <0.001.
